# The role of cholesterol and its oxidation products in tuberculosis pathogenesis

**DOI:** 10.1097/IN9.0000000000000042

**Published:** 2024-04-30

**Authors:** Andrew T. Roth, Jennifer A. Philips, Pallavi Chandra

**Affiliations:** 1 Division of Pulmonary & Critical Care Medicine, Department of Medicine, Washington University School of Medicine, St. Louis, MO, USA; 2 Division of Infectious Diseases, Department of Medicine, Washington University School of Medicine, St. Louis, MO, USA; 3 Department of Molecular Microbiology, Washington University School of Medicine, St. Louis, MO, USA

**Keywords:** *Mycobacterium tuberculosis*, TB, cholesterol, oxysterols, 3β-hydroxysteroid dehydrogenase, cholesterol oxidase, immunometabolism

## Abstract

*Mycobacterium tuberculosis* causes tuberculosis (TB), one of the world’s most deadly infections. Lipids play an important role in *M. tuberculosis* pathogenesis. *M. tuberculosis* grows intracellularly within lipid-laden macrophages and extracellularly within the cholesterol-rich caseum of necrotic granulomas and pulmonary cavities. Evolved from soil saprophytes that are able to metabolize cholesterol from organic matter in the environment, *M. tuberculosis* inherited an extensive and highly conserved machinery to metabolize cholesterol. *M. tuberculosis* uses this machinery to degrade host cholesterol; the products of cholesterol degradation are incorporated into central carbon metabolism and used to generate cell envelope lipids, which play important roles in virulence. The host also modifies cholesterol by enzymatically oxidizing it to a variety of derivatives, collectively called oxysterols, which modulate cholesterol homeostasis and the immune response. Recently, we found that *M. tuberculosis* converts host cholesterol to an oxidized metabolite, cholestenone, that accumulates in the lungs of individuals with TB. *M. tuberculosis* encodes cholesterol-modifying enzymes, including a hydroxysteroid dehydrogenase, a putative cholesterol oxidase, and numerous cytochrome P_450_ monooxygenases. Here, we review what is known about cholesterol and its oxidation products in the pathogenesis of TB. We consider the possibility that the biological function of cholesterol metabolism by *M. tuberculosis* extends beyond a nutritional role.

## 1. Introduction

*Mycobacterium tuberculosis* is one of the world’s most formidable pathogens. It is transmitted person-to-person by an aerosol route and is a master at undermining host immunity, killing more people yearly than any other microbial pathogen ^[1]^. Alveolar macrophages are the first cells productively infected, after which the bacteria disseminate into additional myeloid cells. Macrophages and polymorphonuclear neutrophils are normally dedicated to clearing bacilli, but instead they provide a replicative niche for *M. tuberculosis*. The adaptive immune response, particularly CD4 T cells, is important for establishing well-organized granulomas and preventing bacterial dissemination from the lungs. However, even a robust adaptive immune response fails to reliably sterilize the infection. When host cells in the granuloma die, *M. tuberculosis* grows extracellularly in the cholesterol-rich caseum of necrotic granulomas. Its utilization of host cholesterol, an essential and ubiquitous component of mammalian membranes, is an important element of this strategy. *M. tuberculosis* colocalizes with intracellular lipid droplets within macrophages, establishes lipid inclusions within its own cytoplasm, and depends upon cholesterol uptake to persist during infection ^[2–4]^. *M. tuberculosis* inherited an extensive and highly conserved machinery to metabolize cholesterol, which originated as a way for soil saprophytes to extract organic matter from their environment ^[5–7]^. *M. tuberculosis* retained this machinery, allowing the bacilli to use host cholesterol as a carbon source and to provide precursors for cell envelope lipids that play important roles in virulence ^[4,8]^. *M. tuberculosis* metabolizes cholesterol to an oxidized derivative, called cholestenone. This metabolite was thought to be a required intermediate in cholesterol degradation, but unexpectedly it accumulates during infection of macrophages and in the sputum of people with tuberculosis (TB) ^[9]^. The importance of cholesterol during chronic infection has been attributed to its ability to serve mycobacterial nutritional needs, however, recent studies and the increasing appreciation for the role of oxysterols in immunity point to a potentially broader role for cholesterol and its metabolites on TB pathogenesis. Here, we review what is known about cholesterol and its oxidation products in the pathogenesis of TB and discuss the possibility that bacterial-host cometabolism of cholesterol and oxysterols impacts host immunity.

## 2. Cholesterol fuels *M. tuberculosis* pathogenesis

**Figure 1.**
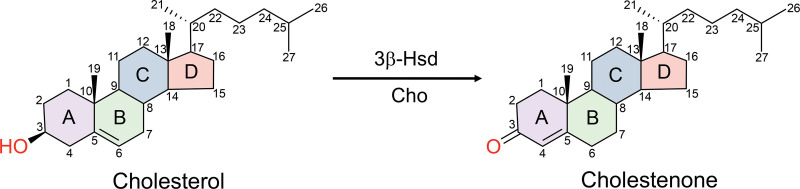
**Enzymatic reaction that converts cholesterol to cholestenone.** 3β-hydroxysteroid dehydrogenases (3β-Hsd) and cholesterol oxidases (Cho) can convert the 3β-hydroxy of cholesterol to a ketone. The core structure of cholesterol is the steroid nucleus, made up of four fused rings that are denoted alphabetically. The carbons are numbered in black.

*M. tuberculosis* cannot synthesize cholesterol, but it encodes an extensive machinery that imports and degrades cholesterol. *M. tuberculosis* isolated from the sputum of individuals with active TB has a transcriptional signature of cholesterol catabolism ^[10,11]^. Most of the cholesterol catabolic genes are clustered together in the genome and are under the control of the tetR-like transcriptional regulators KstR1 and KstR2, which recognize a shared 14 base pair semi-palindromic sequence (reviewed in ref ^[12]^). *M. tuberculosis* first transports sterols across its complex cell envelope and into its cytoplasm using the Mce4 complex ^[4,13]^. Cholesterol is a 27-carbon compound composed of four hydrocarbon rings, which are a feature of all steroid hormones, along with a hydrocarbon side chain (Figure 1). *M. tuberculosis* degrades the side chain primarily through beta-oxidation and also opens the ring structure, catabolizing it to metabolic intermediates (reviewed in ref ^[14]^). Cholesterol catabolism generates acetyl CoA, propionyl CoA, succinyl CoA, and pyruvate, which can be incorporated into central carbon metabolism. While propionyl CoA can enter the tricarboxylic acid cycle, it is also converted to methylmalonyl CoA and shuttled into the synthesis of polyketide virulence lipids in *M. tuberculosis*, such as phthiocerol dimycocerosates (PDIM) and sulfolipid-1 ^[4,8,15,16]^.

Cholesterol catabolism by *M. tuberculosis* appears to be particularly important during chronic infection. An *M. tuberculosis* mutant that is unable to import and utilize cholesterol due to impaired Mce4 complex function is attenuated in mice following approximately 4 weeks of infection, after the onset of adaptive immunity ^[4]^. *M. tuberculosis* that lacks enzymes essential for cholesterol side-chain degradation such as FadA5 exhibits a similar phenotype ^[17]^. One interpretation of these findings is that cholesterol is an essential nutrient during chronic infection. However, *M. tuberculosis* can cometabolize a variety of nutrients to feed central metabolism, including carbohydrates, sugars, fatty acids, amino acids, and cholesterol ^[18,19]^. This gives the bacilli considerable flexibility in metabolizing different combinations of nutrients that it encounters during infection. Why the nutritional environment becomes more constrained, with a greater dependence on cholesterol during chronic infection, is not clear. An alternative possibility is that bacterial cholesterol metabolism is involved in altering host-pathogen interactions in ways that become particularly important during chronic infection.

Additional evidence supporting the importance of cholesterol in *M. tuberculosis* pathogenesis comes from the use of statins. Statins are a group of medications that inhibit HMG-CoA reductase, which mediates the rate-limiting step of cholesterol biosynthesis and is also required for isoprenoid production. Statins have pleiotropic effects, including immunomodulatory and anti-inflammatory effects. Retrospective cohort studies found a reduced incidence of active TB in people prescribed statins ^[20,21]^. While there are many possible explanations for findings from retrospective studies, Parihar et al ^[22]^ demonstrated that mice treated with statins prior to infection have a lower *M. tuberculosis* burden than untreated mice. Subsequent studies in murine bone marrow-derived macrophages and human macrophage cell lines found that statins promote the ability of macrophages to clear *M. tuberculosis* ^[22–24]^. Rescue experiments suggested that at least part of the impact of statins on the antimicrobial activity of macrophages was related to impaired cholesterol synthesis ^[22]^. In addition, when a broad range of statins were used in combination with standard four-drug antibiotic therapy, *M. tuberculosis*-infected mice had improvement in bactericidal activity compared to mice treated with standard antibiotic therapy alone ^[23]^. The extent to which the antimycobacterial activity of statins in vivo is related to their impact on cholesterol remains to be determined, but these studies set the stage for the Phase 2B StAT-TB dose-finding clinical trial (NCT03882177) investigating the safety, tolerability, and pharmacokinetics of pravastatin as adjunctive therapy in participants with drug-susceptible pulmonary TB. Additionally, the open-label ATORTUB clinical trial (NCT04721795) randomized participants to receive standard-of-care antibiotic therapy with or without atorvastatin. Following 2 months of treatment, patients randomized to receive atorvastatin had an increased rate of sputum culture conversion and reduced chest x-ray severity scores, demonstrating the potential of targeting cholesterol as host-directed therapy ^[25]^.

## 3. Host plasma membrane cholesterol: a bacterial target

Cholesterol is essential to mammalian membranes because it maintains their physical and structural properties and supports the organization of microdomains known as lipid rafts. In the plasma membrane, cholesterol condenses the packing of saturated hydrocarbon chains of sphingolipids in the exoplasmic leaflet. Receptors that cluster in lipid rafts are associated with vital cellular processes including endocytosis, exocytosis, receptor trafficking, and cell signaling. Pathogens take advantage of cholesterol availability in membranes to manipulate host cell biology and facilitate pathogenesis. For example, many bacterial pathogens, including *Listeria monocytogenes*, *Streptococcus pneumoniae*, and *Clostridium perfringens*, use cholesterol as a cellular receptor for cholesterol-dependent cytolysins (reviewed in ref ^[26]^). Members of this large family of pore-forming toxins are secreted from bacteria as monomers and bind to cholesterol-rich membranes, which triggers their oligomerization and subsequent assembly into membrane-spanning pores. Recent work demonstrates that the accessibility of host cholesterol in the plasma membrane is also critical in facilitating cell-to-cell spread and intracellular dissemination of the bacterial pathogens *L. monocytogenes* and *Shigella flexneri* ^[27]^. Interestingly, proinflammatory signals alter plasma membrane cholesterol composition, as discussed below, perhaps as a way to defend against these bacterial toxins ^[28]^. While *M. tuberculosis* does not encode cholesterol-dependent cytolysins, the *M. tuberculosis* cell wall virulence lipid PDIM intercalates into host membranes in a manner that depends on the cholesterol content and is reduced by statin treatment ^[29]^. In addition, host plasma membrane cholesterol may impact mycobacterial uptake and potentially intracellular survival, although reports differ ^[30,31]^. Thus, like many other pathogens, *M. tuberculosis* takes advantage of host plasma membrane cholesterol.

## 4. Cholesterol homeostasis and oxysterol production

**Figure 2.**
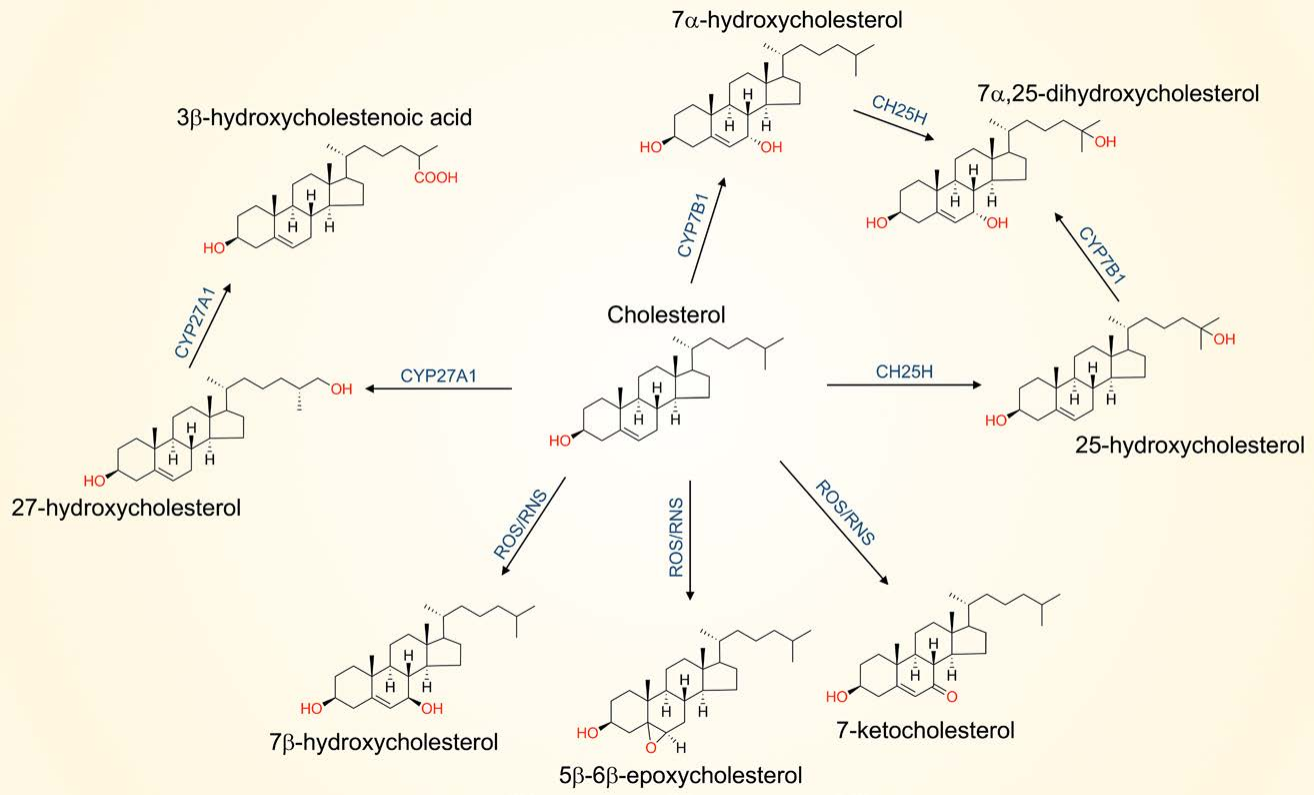
**Synthesis of selected oxysterols within the lung.** Cholesterol can be converted to 27-hydroxycholesterol by CYP27A1 present in the inner mitochondrial membrane. Further oxidation of 27-hydroxycholesterol by CYP27A1 generates 3β-hydroxycholestenoic acid which is important in reverse cholesterol transport from the lung. Cholesterol 25-hydroxylase (CH25H) is localized to the endoplasmic reticulum and converts cholesterol to 25-hydroxycholesterol. It is expressed in myeloid and lymphoid cell types within the lung. CYP7B1, also localized to the endoplasmic reticulum, catalyzes conversion of cholesterol to 7α-hydroxycholesterol. Further oxidation of 7α-hydroxycholesterol by CH25H or 25-hydroxycholesterol by CYP7B1 generates 7α,25-dihydroxycholesterol, which is a principal agonist of GPR183. The oxysterols 7β-hydroxycholesterol, 5β-6β-epoxycholesterol, and 7-ketocholesterol can be produced from cholesterol by reactive oxygen species or reactive nitrogen species (ROS/RNS) and are found in inflammatory disease states.

Humans take up cholesterol from their diet, synthesize it, and modify it to bioactive metabolites. Cholesterol content within cells is maintained largely through a balance between pathways regulated by the transcription factors sterol regulatory element-binding protein-2 (SREBP-2) and liver X receptors (LXRs) ^[32]^. Unlike *M. tuberculosis*, humans are not able to degrade cholesterol. Instead, excess cholesterol is esterified and stored within lipid bodies, transported to the liver via lipoproteins for secretion in bile, or converted to other metabolites including steroid hormones and vitamin D ^[32]^. Cholesterol is also oxidized to derivatives collectively called oxysterols (when we refer to oxysterols, we are including acidic oxysterols, ie, cholestenoic acids and derivatives). Structures of selected oxysterols are shown in Figure 2. Oxysterols such as 27-, 24-, 25-, and 7α-hydroxycholesterol are largely generated enzymatically by members of the cytochrome P_450_ family, the hydroxysteroid dehydrogenase (HSD) family, or diiron cofactor-dependent enzymes such as cholesterol 25-hydroxylase (CH25H) ^[33]^. A minor subset of oxysterols can also be generated nonenzymatically such as 7-ketocholesterol, 7β-hydroxycholesterol and 5β,6β-epoxycholesterol and are associated with cytotoxicity and pathological conditions such as atherosclerosis ^[34,35]^. In general, the addition of oxygen groups to cholesterol, including hydroxyls, carbonyls, and epoxides, increases its hydrophilicity and enhances excretion through bile acids. Studies from the 1970s demonstrated that oxysterols regulate lipid metabolism ^[36,37]^, and since then, there has been a growing appreciation for the roles of oxysterols in cholesterol homeostasis and immunity ^[38]^.

## 5. Oxysterols and immunity

**Figure 3.**
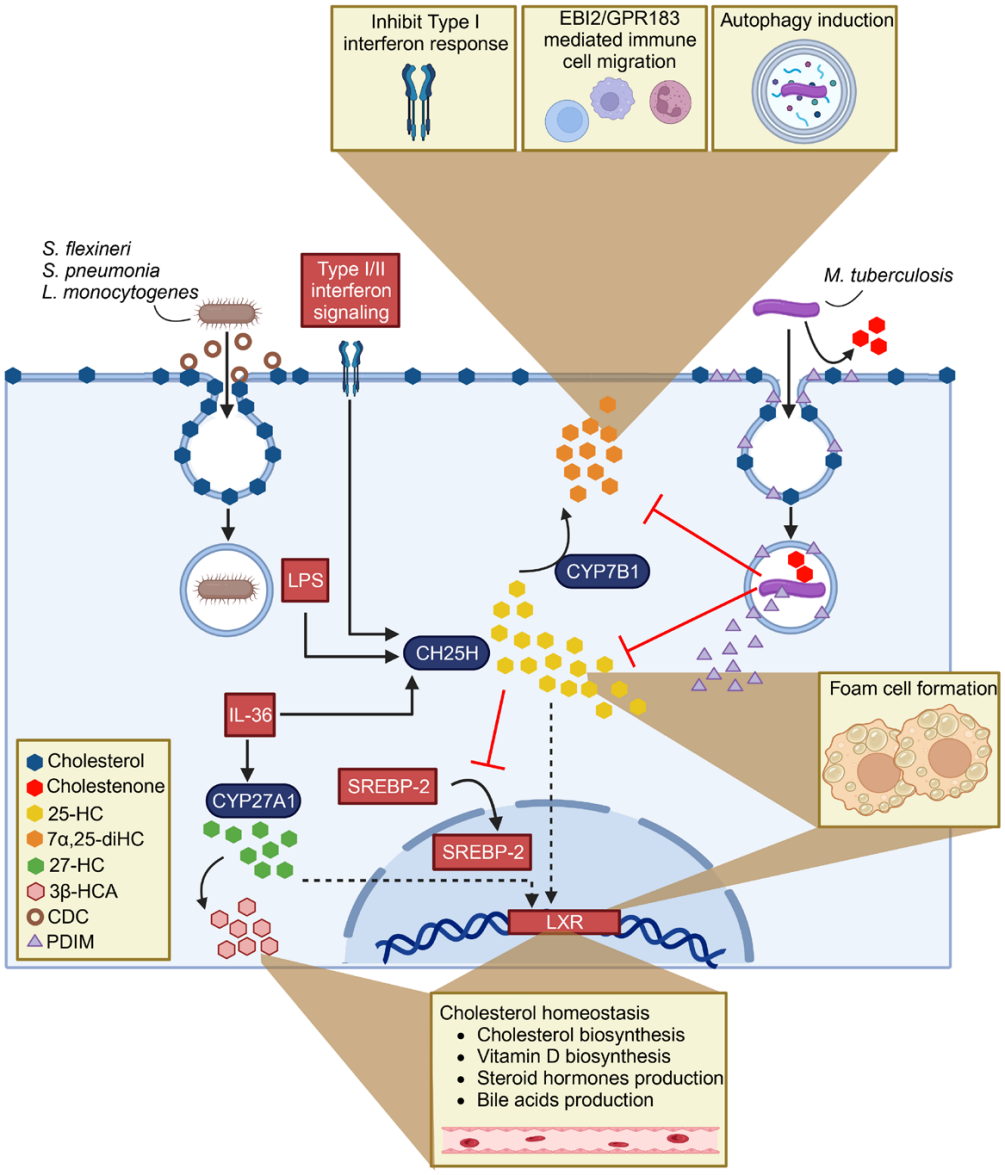
**Illustration depicting the relationship between host oxysterols and infection with *M. tuberculosis.*** Cholesterol-dependent cytolysins (CDCs) from bacterial pathogens such as *S. flexneri*, *S. pneumoniae*, and *L. monocytogenes* form pores in cholesterol-rich membranes. PDIM is shed from the *M. tuberculosis* envelope and intercalates in host membranes in a cholesterol-dependent manner. *M. tuberculosis* oxidizes cholesterol to cholestenone. Pathogen-associated molecular patterns such as LPS and inflammatory stimuli, such as IL-36, and type I and II interferon signaling, trigger the production of immunoactive oxysterols. 25-hydroxycholesterol (25-HC), 27-hydroxycholesterol (27-HC), 7α, 25-dihydroxycholesterol (7α,25-diHC), and 3β-hydroxycholestenoic acid (3β-HCA) can influence important pathways during mycobacterial infection such as foam cell formation, autophagy, immune cell recruitment, and cholesterol homeostasis. Red inhibitor arrows reflect the possibility that *M. tuberculosis* could modify host oxysterols. LPS, lipopolysaccharide; PDIM, phthiocerol dimycocerosates.

Oxysterols are generated in the lungs and play important roles in cholesterol homeostasis and immunity. They are, therefore, likely relevant in the context of *M. tuberculosis*, although there are few studies investigating their role during TB infection. One of the best-studied oxysterols is 25-hydroxycholesterol (25-HC). Prior to the discovery of CH25H, 25-HC was thought to be produced nonenzymatically since it was the most abundant oxysterol detected when cholesterol was stored long-term. However, this possibility was reevaluated when it was found that oxidation of low-density lipoproteins (LDLs) produced limited 25-HC compared to other nonenzymatically produced oxysterols such as 7-ketocholesterol ^[39]^. Thus, in vivo 25-HC is thought to mainly be derived enzymatically or through dietary sources. In alveolar macrophages, CH25H catalyzes the formation of 25-HC in response to inflammatory stimuli, including type I and II interferons, and pathogen-associated molecular patterns such as lipopolysaccharide (LPS) (Figure 3) ^[40,41]^. As would be anticipated, 25-HC is induced in the lungs of mice infected with *M. tuberculosis* ^[42]^. 25-HC promotes intracellular cholesterol accumulation both by downregulation of sterol regulatory element-binding protein-2 processing as well as through agonism of LXRs ^[38]^. As such, it provides a direct link between inflammatory responses and cholesterol regulation. The proinflammatory cytokine IL-36 also induces CH25H, as well as CYP27A1, to generate 25-HC and 27-HC, which are both LXR ligands ^[43]^. LXRα confers resistance to *M. tuberculosis*, promoting neutrophil recruitment early during infection and enhancing T_H_1/T_H_17 functions in the lungs ^[44]^. CYP27A1 also further oxidizes 27-HC to 3β-hydroxycholestenoic acid, which contributes to reverse cholesterol transport out of the lung. 3β-hydroxycholestenoic acid is notable as the majority of serum levels of this oxysterol are derived from biosynthesis in the lung ^[45]^. Recent studies have shown a crucial role for 25-HC in reducing susceptibility to cholesterol-dependent cytolysins and dampening the intraepithelial spread of bacterial pathogens such as *L. monocytogenes* and *S. flexneri* ^[27,46]^. The proposed mechanism suggests that 25-HC triggers the rapid mobilization of “accessible” plasma membrane cholesterol, which is not bound to sphingomyelin, and sequesters it intracellularly in lipid droplets as cholesterol esters ^[27,46,47]^. Interestingly, while 25-HC appears to be protective against *L. monocytogenes* infection when secreted in a paracrine fashion, macrophages treated with 25-HC exhibited an increased bacterial burden ^[27]^. This suggests that there are important distinctions in the effects of 25-HC, whether paracrine or autocrine, on host defense mechanisms ^[27]^. In addition, in vivo 25-HC was shown to resolve lung inflammation by promoting the resolution of neutrophilia through enhanced efferocytosis ^[41]^. Thus, 25-HC could potentially impact *M. tuberculosis* pathogenesis in a variety of ways: by reducing plasma membrane accessible cholesterol, promoting efferocytosis, enhancing T_H_1/T_H_17 functions, and/or increasing intracellular lipids. While the impact of 25-HC on *M. tuberculosis* has not been directly investigated, a recent study found that 25-HC promotes foam cell formation and enhances bacterial burden in a mouse model of *Mycobacterium marinum* infection, and those effects were dependent on bacterial production of PDIM ^[48]^. Interestingly, *M. tuberculosis* encodes at least three enzymes belonging to the cytochrome P_450_ family (Cyp124, Cyp125, and Cy142) that are capable of hydroxylating the terminal carbon in the cholesterol side chain. In doing so, *M. tuberculosis* could also generate LXR agonists ^[49–51]^.

25-HC can be further hydroxylated by the host enzyme CYP7B1 into 7α,25-dihydroxycholesterol (7α,25-diHC), which is a potent ligand for the G-protein coupled receptor, Epstein-Barr virus-induced gene 2 (EBI2 or GPR183). 7α,25-diHC directs immune cell migration through GPR183, including directing B and T cell migration within the lymph node ^[52–54]^. 7α,25-diHC-mediated activation of GPR183 was shown to enhance uptake and constrain intracellular replication of *M. tuberculosis* within human monocytes ^[55]^. During SARS-CoV-2 and Influenza A virus infection, 7α,25-diHC drives macrophage infiltration into the lungs ^[56]^. Following infection with *M. tuberculosis*, mice with diet-induced dysglycemia had reduced lung expression of *Cyp7b1* and *Gpr183,* accompanied by impairment in macrophage migration to the lungs and reduced control of infection. This suggests that 7α,25-diHC is also critical for the migration of macrophages to the site of *M. tuberculosis* infection ^[42]^. GPR183 activation was recently shown to confer protection against *M. tuberculosis* by inducing autophagy, dampening type I IFN responses, and mediating early eosinophil recruitment to the lungs ^[55,57]^. Interestingly, 7α,25-diHC is inactivated by 3β-oxidation ^[54]^, and the *M. tuberculosis* enzyme 3β-Hsd can carry out this reaction, as discussed below. In summary, there is an increasing understanding of the antimicrobial and immunometabolic functions of oxysterols. While further studies are required to understand their impact on *M. tuberculosis* pathogenesis, it is tempting to speculate that *M. tuberculosis* manipulates oxysterol metabolism to its advantage.

## 6. Cholesterol oxidases of *M. tuberculosis*

*M. tuberculosis* and other Actinobacteria can degrade cholesterol. In the Actinobacteria where it was first studied, the initial step in cholesterol catabolism was shown to require the conversion of the 3β-hydroxyl at the third carbon position of cholesterol to a ketone and the subsequent Δ^5^–Δ^4^ isomerization of cholesterol, leading to the production of cholest-4-en-3-one (cholestenone) (Figure 1). We found that cholestenone accumulates over time in *M. tuberculosis*-infected macrophages and is significantly elevated in the sputum of individuals with TB compared to individuals who have TB-like symptoms for other reasons ^[9]^. Previously, it was thought that *M. tuberculosis*, like other Actinobacteria, produces cholestenone as a requisite intermediate in cholesterol degradation ^[12,14]^. However, we found that *M. tuberculosis* can utilize cholesterol even without making cholestenone ^[9]^, raising the question as to the function of cholestenone and the enzymes that make it.

The conversion of cholesterol to cholestenone can be carried out by two evolutionarily unrelated enzyme families, 3β-hydroxysteroid dehydrogenases and cholesterol oxidases. *M. tuberculosis* encodes a member of both families. 3β-hydroxysteroid dehydrogenases are members of the short-chain alcohol dehydrogenase superfamily and are represented in both humans and microbes ^[58]^. Cholesterol oxidases belong to the glucose methanol choline oxidoreductase superfamily of enzymes and, in contrast to 3β-hydroxysteroid dehydrogenases, are almost exclusively found in bacteria. *M. tuberculosis* 3β-Hsd is encoded by *hsd/Rv1106c*. Recombinant 3β-Hsd can convert cholesterol to cholestenone ^[49,59]^. When *hsd* is deleted from *M. tuberculosis*, conversion of cholesterol to cholestenone is markedly impaired, both when the bacilli grow in liquid media supplemented with cholesterol and when growing in macrophages ^[9,59]^. The *M. tuberculosis* enzyme ChoD (encoded by *choD*/Rv3409c) is annotated as a cholesterol oxidase. These flavoenzymes couple the oxidation of cholesterol to the reduction of molecular oxygen, creating H_2_O_2_ as a product. In *Rhodococcus hoagii* (previously *Rhodococcus equi*) and *Streptomyces*, the ChoE cholesterol oxidase is required for the utilization of cholesterol as a carbon source. Although annotated as a cholesterol oxidase, *M. tuberculosis* ChoD shares limited homology with other cholesterol oxidases and definitive evidence that is a cholesterol oxidase is lacking. Yang et al ^[60]^ were unable to demonstrate cholesterol oxidase activity from the *Mycobacterium smegmatis* ChoD homolog (MSMEG_1604). When we deleted *choD* from *M. tuberculosis*, there was no impact on cholestenone production in vitro, but *choD* is known to have markedly reduced expression under in vitro relative to in vivo conditions ^[61]^. Other studies support ChoD as a functional cholesterol oxidase. Brzostek et al ^[62]^ found that when an *M. smegmatis* strain engineered to express *M. tuberculosis* ChoD was grown in cholesterol, there was increased cholestenone detected in the supernatant relative to the control strain. In addition, when *M. tuberculosis* ChoD was expressed in *E. coli*, H_2_O_2_ was detected, in support of cholesterol oxidase activity ^[62]^. Thus, 3β-Hsd and potentially ChoD of *M. tuberculosis* may both be capable of producing cholestenone.

In addition to both *hsd* and *choD* being dispensable for cholesterol utilization ^[63]^, the genomic location and regulation of *hsd* and *choD* argue for roles beyond cholesterol catabolism ^[64]^. First, these enzymes are not encoded in the “Cho region,” which contains most of the cholesterol-degrading machinery of *M. tuberculosis* (reviewed in ref ^[12]^). Secondly, as mentioned above, cholesterol catabolism in *M. tuberculosis* and other mycobacteria is controlled by KstR binding to a conserved operator region just upstream to the transcriptional start site of the regulated genes ^[65,66]^. There is an operator proximal to *hsd* in most mycobacterial species, including *M. marinum*, *Mycobacterium leprae,* slow-growing nontuberculous mycobacteria, and rapidly growing mycobacteria. However, there is no KstR-binding site upstream of *hsd* in members of the *M. tuberculosis* complex. This suggests that it was advantageous for *hsd* to be transcriptionally decoupled from cholesterol and lipid catabolism in *M. tuberculosis* ^[66]^. In support of this, *hsd* expression in *M. tuberculosis* is not induced by cholesterol ^[17]^. *choD (Rv3409c*) and its orthologous genes including *MSMEG_1604* also are not regulated by KstR1 or KstR2 ^[64]^. A third piece of evidence that these enzymes play roles outside of cholesterol catabolism is from *M. leprae*, which has undergone significant reductive evolution relative to other mycobacteria. *M. leprae* lost the ability to degrade cholesterol, yet retains *hsd, choD*, and the ability to make cholestenone ^[67,68]^.

There are limited in vivo data on the role of *hsd* and *choD* in *M. tuberculosis* infection. Following aerosol infection of guinea pigs, there was no significant difference in bacterial burden between animals infected with wild type or ∆*hsd M. tuberculosis*, however, the animals infected with ∆*hsd* had a greater number of lung granulomas, suggesting that it might influence the inflammatory response ^[60]^. The phenotype of *choD* mutants in vivo has only been reported for a strain of *M. tuberculosis* (H37Ra) that is already highly attenuated. The ∆*choD* H37Ra strain was more highly attenuated in peritoneal macrophages as well as in mice after intravenous infection compared to the parent H37Ra strain ^[62]^. Several in vitro experiments point to the role of ChoD in modulating the mycobacterial surface. The ∆*choD* mutant in virulent *M. tuberculosis* is attenuated in macrophages, eliciting higher levels of reactive nitrogen and oxygen species in a manner that depends upon toll-like receptor 2. A transposon mutant affecting the orthologous protein in *M. smegmatis* (MSMEG_1604) failed to acetylate a surface glycopeptidolipid ^[69]^. Thus, although the data are limited, the phenotypes of ∆*hsd* and ∆*choD M. tuberculosis* appear different than the phenotype associated with cholesterol uptake and degradation defects. To conclude, how 3β-Hsd and ChoD impact *M. tuberculosis* pathogenesis is not well understood.

## 7. Cholestenone: beyond bacterial nutrition?

Bacteria use cholesterol oxidases in diverse ways that may offer insight into non-nutritional roles for cholesterol oxidation during *M. tuberculosis* infection. By increasing the ratio of cholestenone to cholesterol in host membranes, cholesterol oxidases modify membrane fluidity, disrupting lipid rafts and impacting crucial processes such as receptor signaling and vesicular integrity ^[70–74]^. Cholesterol oxidase can synergize with sphingomyelinases that liberate sphingomyelin-bound cholesterol to cause hemolysis of red blood cells ^[75,76]^. The membrane-disrupting activity of cholesterol oxidases has larvicidal effects that have been utilized in the agricultural industry ^[74]^. To directly investigate the effects of cholestenone on membrane structure, Neuvonen et al ^[70]^ treated primary human fibroblasts with recombinant cholesterol oxidase and found that membrane enriched in cholestenone had decreased order. Furthermore, cholestenone was more readily desorbed from the plasma membrane by lipoproteins and other extracellular acceptors relative to cholesterol. Interestingly, a mutant cholesterol oxidase with impaired isomerase activity that produced cholest-5-ene-3-one as its major product did not disrupt membrane permeability, suggesting that the isomerization reaction also impacts membrane function ^[73]^.

The obligate intracellular pathogen *M. leprae* provides important insights given that it performs cholesterol 3β-oxidation, but not cholesterol catabolism. While the genes involved in cholesterol catabolism are lost or exist as pseudogenes, *M. leprae* maintains orthologues to *M. tuberculosis* 3β-Hsd (ML1942) and ChoD (ML0389). Rosa et al demonstrated that *M. leprae* uses 3β-HSD to convert cholesterol to cholestenone and that *M. leprae* lacking ML1942 is attenuated during infection of Schwann cells ^[67]^. They further showed that this NADH-generating reaction is an important source of electrons used for ATP generation and maintenance of intracellular infection ^[67]^. Cholesterol oxidases have also been shown to play a regulatory role in bacteria. The *pimE* gene of *Streptomyces natalensis* encodes a functional cholesterol oxidase that is an essential signaling molecule required for bacterial production of the antifungal polyene antibiotic pimaricin ^[77]^. Cholestenone is not toxic to *M. tuberculosis* ^[9]^. however, it does inhibit the growth of *Helicobacter pylori,* suggesting cholestenone may enhance competitive exclusion in certain contexts ^[78]^. To conclude, microbes utilize cholesterol 3β-oxidation for a variety of purposes.

## 8. Hsd function: beyond cholesterol oxidation?

The broad substrate specificity of *M. tuberculosis* 3β-Hsd supports the possibility that it modifies additional steroids or oxysterols. Steroid hormones are generated from cholesterol after side-chain cleavage. Recombinant 3β-Hsd from *M. tuberculosis* can efficiently catalyze the 3β-oxidation of pregnenolone to progesterone and dehydroepiandrosterone to androsterone, two key intermediates in the production of glucocorticoids and sex steroids ^[59]^. Varaksa et al ^[49]^ used a spectrophotometric titration assay to evaluate the affinity of sterols to the active site of *M. tuberculosis* 3β-Hsd and found that 25-HC, 7α-hydroxycholesterol, and 7α,25-diHC were among its substrates. 7α,25-diHC had the highest turnover of the substrates tested. This is of particular interest since it is a potent activator of GPR183, and 7α,25-diHC is inactivated by 3β-oxidation ^[54]^. Interestingly, mycobacterial species that are members of the microbiome may modulate human steroid levels. Li et al ^[79]^ found that *Mycolicibacterium (Mycobacterium) neoaurum* was overrepresented in the gut microbiome of a cohort of patients with depression associated with low testosterone. Moreover, the *M. neoaurum* gene *MN2019_09805* oxidized testosterone to androstenedione via 17β oxidation. MN2019_09805 is an ortholog of *M. tuberculosis* 3β-Hsd with ~74% protein similarity and is arranged in the same four-gene operon structure seen in *M. tuberculosis*.

## 9. Conclusions

The pathogenesis of *M. tuberculosis* is intimately linked to host fatty acid and cholesterol utilization. *M. tuberculosis* is dependent on host lipids to sustain infection, induces foamy macrophage formation, and has dedicated pathways for cholesterol import and degradation ^[2,4,80,81]^. Despite the close relationship between *M. tuberculosis* and cholesterol, we have a relatively limited understanding of how cholesterol and its derivatives impact TB disease. The accumulating scientific evidence demonstrating the impact of oxysterols on immunity makes them important candidates for therapeutic interventions. However, the overall outcome of modulating oxysterols in any infectious context is likely to be complex given their direct antimicrobial and immunometabolic effects. During TB, the importance of cholesterol has largely been attributed to its ability to serve mycobacterial nutrition, however, the increasing appreciation for the role of oxysterols in immunity points to a potentially broader role for cholesterol cometabolism and its effects on TB pathogenesis. We found, unexpectedly, that cholestenone is not a requisite intermediate in cholesterol degradation, and that *M. tuberculosis* infection leads to the accumulation of cholestenone in people ^[9]^. The function of this metabolite during infection remains to be established. In addition, *M. tuberculosis* 3β-Hsd can metabolize other oxysterols such as 25-HC and 7α,25-diHC ^[49]^, potentially altering the host immune response and cholesterol homeostasis during infection. Beyond 3β-Hsd*, M. tuberculosis* ChoD, Cyp124, Cyp125, and Cyp142 may metabolize cholesterol, oxysterols, or steroids ^[49]^. Understanding the interplay between host and *M. tuberculosis* oxysterol modifications will require rigorous identification of infection-induced metabolites, establishing the biosynthetic pathways leading to their production, and characterizing their impact on disease outcome. This knowledge will lend fundamental insight into one of the world’s most deadly pathogens and may facilitate the development of adjunct therapies and biomarkers for TB.

## Author contributions

A.T.R., J.A.P., P.C. wrote and edited the manuscript. All authors have read and approved the final manuscript.

## Conflicts of interest

The authors declare no conflict of interest.

## Funding

This work was supported by the National Institutes of Health: R21AI160386, R01AI178685, and R01AI30454 to J.A.P., and the Washington University Institute of Clinical and Translational Sciences grant UL1TR002345 from the National Center for Advancing Translational Sciences (NCATS). The content is solely the responsibility of the authors and does not necessarily represent the official view of the NIH. A.T.R. holds a Dean’s Scholars Award from the Washington University Division of Physician-Scientists, which is funded by a Burroughs Wellcome Fund Physician-Scientist Institutional Award.

## Acknowledgments

Figures were created with Biorender using original templates. ChemDraw 23.0.1 was used to prepare molecular structures.
